# Organization-Level Factors Associated with Changes in the Delivery of the Five A’s for Smoking Cessation following the Implementation of a Comprehensive Tobacco-Free Workplace Program within Substance Use Treatment Centers

**DOI:** 10.3390/ijerph191911850

**Published:** 2022-09-20

**Authors:** Cassidy R. LoParco, Tzuan A. Chen, Isabel Martinez Leal, Maggie Britton, Brian J. Carter, Virmarie Correa-Fernández, Bryce Kyburz, Teresa Williams, Kathleen Casey, Anastasia Rogova, Hsien-Chang Lin, Lorraine R. Reitzel

**Affiliations:** 1 Department of Health Behavior and Health Systems, School of Public Health, University of North Texas Health Science Center, 3500 Camp Bowie Blvd, Fort Worth, TX 76107, USA; 2 Department of Psychological, Health, and Learning Sciences, University of Houston, 3657 Cullen Blvd Stephen Power Farish Hall, Houston, TX 77204, USA; 3 HEALTH Research Institute, University of Houston, 4849 Martin Luther King Blvd., Houston, TX 77204, USA; 4 Integral Care, 1430 Collier Street, Austin, TX 78704, USA; 5 Department of Applied Health Science, School of Public Health, Indiana University-Bloomington, 1025 E. 7th St., Bloomington, IN 47405, USA

**Keywords:** tobacco control, behavioral health, provider self-efficacy, smoking, brief intervention, smoking cessation, workplace program, substance use treatment center, barriers, implementation science

## Abstract

Many adults with a substance use disorder smoke cigarettes. However, tobacco use is not commonly addressed in substance use treatment centers. This study examined how provider beliefs about addressing tobacco use during non-nicotine substance use treatment, provider self-efficacy in delivering tobacco use assessments, and perceived barriers to the routine provision of tobacco care were associated with changes in the delivery of the evidence-based five A’s for smoking intervention (asking, advising, assessing, assisting, and arranging) at the organizational level. The data were from 15 substance use treatment centers that implemented a tobacco-free workplace program; data were collected before and after the program’s implementation. Linear regression examined how center-level averages of provider factors (1) at pre-implementation and (2) post- minus pre-implementation were associated with changes in the use of the five A’s for smoking in substance use treatment patients. The results indicated that centers with providers endorsing less agreement that tobacco use should be addressed in non-nicotine substance use treatment and reporting lower self-efficacy for providing tobacco use assessments at pre-implementation were associated with significant increases in asking patients about smoking, assessing interest in quitting and assisting with a quit attempt by post-implementation. Centers reporting more barriers at pre-implementation and centers that had greater reductions in reported barriers to treatment over time had greater increases in assessing patients’ interest in quitting smoking and assisting with a quit attempt by post-implementation. Overall, the centers that had the most to learn regarding addressing patients’ tobacco use had greater changes in their use of the five A’s compared to centers whose personnel were already better informed and trained. Findings from this study advance implementation science and contribute information relevant to reducing the research-to-practice translational gap in tobacco control for a patient group that suffers tobacco-related health disparities.

## 1. Introduction

Although the prevalence of cigarette smoking has been decreasing in the U.S. since 2005, around 13% of the adult population still smokes [1]. Adults with non-nicotine substance use disorders, however, smoke at rates nearly five to seven times greater than the general U.S. adult population [1,2]. Specifically, 65–87% of adults with a substance use disorder smoke cigarettes, and they tend to smoke more heavily when compared to adults without a substance use disorder [1,2]. Smoking is causally related to an increased risk of several chronic diseases, including various cancers, heart and lung disease, and stroke [3]. Importantly, smoking is responsible for approximately half a million deaths annually in the U.S. and is the number one preventable cause of morbidity and mortality [4]. Moreover, mortality among individuals with substance use disorders tends to largely be attributed to their smoking rather than to the use of a non-nicotine substance [5,6].

Many people who smoke want to quit, including individuals with a substance use disorder [7,8,9,10]. Only a small percentage of individuals who smoke successfully quit each year [11,12,13,14,15]. Moreover, tobacco use is not commonly addressed in substance use treatment centers among individuals with a non-nicotine substance use disorder. For example, a previous U.S. national study found that less than half of substance use treatment centers offered smoking cessation counseling or nicotine replacement therapy [16]. This is a missed opportunity for improving patient care given that smoking cessation promotes substance use disorder treatment, recovery, and reduces the substance use disorder relapse risk [17,18,19,20]. Given the high rates of smoking among substance use disorder patients, there is a critical need for substance use treatment centers to build their capacity to address patients’ smoking in conjunction with their non-nicotine substance use needs.

Research demonstrates several reasons why smoking cessation is not typically addressed within non-nicotine substance use treatment center settings. For example, providers may be concerned that addressing their patients’ smoking may interfere with their non-nicotine substance use recovery [21]. Additionally, a variety of other barriers exist, including a lack of knowledge and training in treating tobacco dependence (i.e., low self-efficacy), inadequate time and resources, and the high rates of smoking among the providers themselves [22,23,24,25,26,27,28,29,30,31,32,33,34,35,36,37,38].

The implementation of comprehensive tobacco-free workplace programs in substance use treatment centers may help bridge the research-to-practice translation gap for smoking cessation care provision in these settings. Comprehensive tobacco-free workplace programs reduce exposure to tobacco smoke and encourage smoking abstinence by prohibiting tobacco use on-site, including indoors and on all center property [39,40,41,42]. Tobacco-free workplace programs also provide resources and tobacco-related education to employees, address provider misconceptions about treating tobacco use during non-nicotine substance use disorder recovery, increase provider self-efficacy by training how to screen patients for tobacco use and address their smoking, and change organizational norms about tobacco use [42,43,44,45,46]. An intervention component commonly included within these programs is instruction on the use of the five A’s [47,48,49]. The five A’s are widely recognized as the standard for a successful tobacco use treatment intervention and include five components: (1) ask patients about their smoking, (2) advise them to quit, (3) assess their willingness to quit, (4) assist in their quitting via treatment or referral, and (5) arrange a follow-up [47,48,49,50,51,52].

Comprehensive tobacco-free workplace programs have been successfully implemented in substance use treatment centers previously, increasing employee knowledge about smoking cessation and changing providers’ behaviors to more routinely screen for and provide smoking cessation services to their patients [45,52,53,54,55]. However, little is known about how broader-level influences (e.g., center-level provider factors) can impact the success of these tobacco-free workplace programs by influencing providers’ behaviors within the center overall (e.g., increasing the provision of tobacco cessation interventions). Several prominent implementation science frameworks and multilevel models of health behavior, including, but not limited to, the Socioecological Model [56], suggest the importance of broader-level factors (e.g., organization-level factors such as normative beliefs, values, culture, and policies and/or communities of practice among providers [57]) in influencing the overall patient care provision. Understanding more about the impact of center-level factors in the context of a comprehensive tobacco-free workplace program can help identify important predictors of desirable provider behavior changes within substance use treatment centers.

The current study used data from a comprehensive tobacco-free workplace program that was implemented within substance use treatment centers in Texas, U.S. [45,52,53,54,55]. This program demonstrated statistically significant increases in providers’ provision of four of the five A’s from pre- to post-implementation; assessing patients’ interest in quitting increased but was not significantly changed over the course of the program [52]. Herein, we examine how the following factors were associated with changes in the delivery of the five A’s from pre- to post-program implementation: (1) providers’ beliefs that tobacco use should be addressed during patients’ substance use treatment, (2) providers’ self-efficacy in the delivery of tobacco use assessments, and (3) providers’ perceived number of barriers to the provision of routine tobacco use care. Specifically, these three factors were examined (1) at pre-implementation and (2) as they changed between pre- and post-implementation (post- minus pre-implementation values) to see how they were associated with changes in the five A’s over time. The findings from the current study are important, as they inform the field of implementation science, providing information that may enhance intervention effectiveness in future iterations of workplace program implementation [58,59].

## 2. Materials and Methods

### 2.1. Participants and Recruitment

Participants in the parent study were the employee stakeholders (i.e., clinicians and staff) of 15 substance use treatment centers that enrolled and completed a comprehensive tobacco-free workplace program called Taking Texas Tobacco Free (TTTF) from December 2017 to May 2020. Recruitment was largely accomplished via direct email solicitation and word of mouth. Recruitment was limited to the state of Texas due to the funder’s requirements. Participation in TTTF was initiated by each center’s leadership. Program enrollment was ongoing; each center received the same intervention components over a 7.2 to 13.6 (10.96 ± 3.84)-month implementation timeline, depending on each center’s respective capacity as negotiated with their leadership. Participating substance use treatment centers together served over 80,000 patients during almost 300,000 appointments annually. More information about TTTF can be found in prior publications [42,45,52,53,54,55,60,61], and the characteristics of the centers specifically included in the current report can be found in Le et al. (2021) [52].

### 2.2. Program Implementation

The intervention implemented by TTTF was based on the theory of behavioral and organizational change guided by the Socioecological Model [56] and the Social Cognitive Theory [62]. Specifically, TTTF was designed to affect providers’ routine delivery of the 5 A’s by altering their beliefs about tobacco use disorder care provision via education; improving their self-efficacy for intervention via training and practice; and reducing barriers to intervention with patients through education, training, resource provision, and by changing organizational norms about tobacco use (e.g., through workplace policy changes). Thus, TTTF ultimately aimed to change organizational practices around tobacco use by changing provider-level factors, among other things. The IRB at the University of Houston approved all study procedures.

TTTF implementation started with a 60–120-min education session that included a review of the health risks of tobacco use, disparities for patients with substance dependencies, the use of the 5 A’s for screening and treating tobacco use, the importance and beneficial outcomes of treating tobacco use within a substance use dependency treatment, and medications to assist with treating tobacco use disorder. This evidence-based session was delivered by the TTTF staff and targeted all center employees, with the duration and delivery modality (in person or virtual) negotiated with the center leadership. Centers were provided with training slides on these topics to thereafter include within their new employee orientations. Implementation next progressed to the identification of program champion providers at enrolled centers (i.e., staff, typically clinicians or managers who volunteered to oversee program implementation), who the TTTF program sent to an accredited 4- to 5-day certified tobacco treatment specialist training. Program champions and center providers were asked to attend 7-h motivational interview training delivered by the TTTF staff (university faculty members with advanced Motivational Interviewing training). Centers also received a starter kit of nicotine replacement therapies for patients and employees, along with information on how to continue to budget for these products themselves to keep the supply available post-program implementation. The centers each implemented a comprehensive tobacco-free workplace policy on-site, whereby tobacco product use was not allowed indoors or anywhere on the premises. The TTTF program designed and provided signage to support these efforts and worked closely with the centers to ensure policy monitoring and enforceability. Finally, the centers selected or worked with TTTF staff to tailor passive dissemination materials to the specific characteristics of their populations (e.g., age, sex, and ethnicity) that could be displayed on the patient treatment room walls or taken home by the patients. These materials provided information on the tobacco use among individuals with substance dependencies and described how they can receive care for their tobacco use disorder. The centers were not compensated monetarily for their participation in the TTTF program.

### 2.3. Survey Procedures

An online survey was distributed to the providers (i.e., clinicians with direct patient contact and the employee group of relevance in the current study) within each center by a center representative (usually a program champion) via email at two timepoints. The first timepoint was following center enrollment and was prior to program implementation (known as pre-implementation), and the second timepoint was following the implementation of all program components (known as post-implementation). Each survey was preceded by a cover letter that contained the elements of informed consent, including the purpose of the survey and the voluntary nature of data collection. Data collection was anonymous to maintain provider privacy and encourage honest responding. Moreover, given the known rates of turnover at substance use treatment centers [63,64,65,66] that were anecdotally evident during this program’s implementation, it is likely that some (but unknown exact) proportion of the different providers participated in the pre- versus the post-implementation survey procedures. Providers had about 3 weeks to respond to the survey requests; completion reminders were sent weekly within that timeframe.

### 2.4. Survey Measures

Independent variables. Three survey items comprised the independent variables for this report. Each of the independent variables were assessed pre- and post-implementation. The first item assessed provider beliefs by asking respondents to rate their agreement on a 5-point Likert scale ranging from “strongly agree” to “strongly disagree” to the following statement: “Tobacco use should be addressed during the course of substance use treatment.” The second item assessed provider self-efficacy for tobacco use screening by asking respondents to rate their agreement on a 5-point Likert scale ranging from “strongly agree” to “strongly disagree” to the following statement: “I feel like I am able to effectively deliver tobacco use assessments to patients.” A binary variable was created for each of these 2 items by combining “strongly agree” and “somewhat agree” (1) versus “neither agree nor disagree”, “somewhat disagree”, and “strongly disagree” (0). Finally, the third item assessed perceived barriers by asking providers “What barriers do you face in regularly treating tobacco-using patients? Select all that apply.” The options included: (1) ”I do not know how to treat tobacco use with medications”, (2) ”I do not know how to treat tobacco use with counseling”, (3) ”I do not know where to refer tobacco users for cessation assistance”, (4) ”I do not know how to best motivate tobacco using patients to quit”, (5) ”I believe that tobacco using patients do not want to quit”, (6) ”I believe that tobacco using patients cannot quit”, (7) ”I do not have time to treat tobacco use”, or (8) ”My employer does not want me to treat tobacco use.” Responses to this item were summed, with the concept that more perceived barriers would lead to a greater perceived difficulty in providing care for tobacco use, for a total score ranging from 0 to 8.

Dependent variables. Five survey items representing each of the 5 A’s comprised the dependent variables, each of which were asked pre- and post-implementation. The first item asked providers to self-report whether they asked patients about their smoking status over the preceding month (0 = no, 1 = yes). The other four items asked about whether the provider advised patients to quit smoking, assessed their willingness to make a quit attempt, assisted them in quitting by providing treatment or making a referral for treatment, and arranged to follow up with them to assess their progress regarding smoking cessation. The response options for these items were “yes, all of them”, “yes, some of them”, and “no, none of them”. Similar to previous research [44,52,53,60,67], the responses to each query were coded as no = 0 (“no, none of them”) or yes = 1 (“yes, all of them” and “yes, some of them”).

### 2.5. Statistical Analysis

The analysis unit for this study was the center. First, center-level variables were derived from individual-level (i.e., provider) data for each center at pre- and post- implementation [68,69]. Then, changes in the independent variables and dependent variables were calculated for each center (i.e., %/mean at post-implementation–%/mean at pre-implementation). Linear regression analyses were conducted to explore the associations of (1) pre-implementation independent variable values and (2) changes in each independent variable with the changes in each dependent variable over time. Separate analyses were conducted for each independent/dependent variable pairing. Four centers were excluded in analyses involving provider self-efficacy for tobacco use assessment delivery, as this item was inadvertently not administered to centers enrolled at an early phase of the study. Model assumptions were tested; the results indicated that the assumptions were met. Specifically, the error terms were normally distributed (*p*s > 0.0595), homoscedasticity was met (White tests *p*s = 0.1147–0.9041), and most scatter plots indicated linearity. All analyses were conducted using SAS Version 9.4, and the level of significance was designated at *p* < 0.05.

## 3. Results

### 3.1. Substance Use Center Characteristcs

Of the 15 participating substance use treatment centers, the center-level mean percentage of the pre-implementation variables and the percent changes by post-implementation were as follows: 86.52% believed that tobacco use should be addressed during substance use treatment (+3.90%), 67.98% agreed that they had self-efficacy for effectively delivering tobacco use assessments (+4.14%), 1.60 barriers to routinely providing tobacco use disorder care were endorsed (−0.73), 76.25% endorsed asking (+13.99%), 72.02% endorsed advising (+14.16), 79.80% endorsed assessing (+8.78%), 54.28% endorsed assisting (+26.24%), and 46.23% endorsed arranging (+26.84%) (Table 1).

### 3.2. Provider Belief in Addressing Tobacco Use during Substance Use Treatment

#### 3.2.1. Pre-Implementation

Centers with a lower percent of providers at pre-implementation who believed that tobacco use should be addressed during substance use treatment had greater changes over time (i.e., increases) in asking about patients’ smoking status (B = −0.91, *p* = 0.03) and assisting them in quitting by providing a treatment or making a referral for a treatment (B = −1.52, *p* = 0.01).

#### 3.2.2. Changes (Post- Minus Pre-Implementation)

Despite the increases in the center-level means of beliefs in addressing tobacco use over the course of the intervention, changes in this belief from pre- to post-implementation were not significantly associated with changes over time in the use of the five A’s (Table 2).

### 3.3. Provider Self-Efficacy in Conducting Tobacco Use Assessments

#### 3.3.1. Pre-Implementation

Centers with a lower percent of providers reporting self-efficacy for conducting tobacco use assessments at pre-implementation experienced more changes over time in assessing patients’ willingness to make a quit attempt (B = −0.93, *p* = 0.01).

#### 3.3.2. Changes (Post- Minus Pre-Implementation)

Although the provider self-efficacy for conducting tobacco use assessments increased over time, these changes were not significantly associated with changes over time in the use of the five A’s (Table 2).

### 3.4. Barriers to the Routine Provision of Tobacco Use Disorder Care

#### 3.4.1. Pre-Implementation

Centers reporting more barriers at pre-implementation had greater percent changes over time in assessing patients’ willingness to make a quit attempt (B = 23.40, *p* = 0.01).

#### 3.4.2. Changes (Post- Minus Pre-Implementation)

Centers experiencing greater decreases in barriers over time were associated with greater increases in assessing patients’ willingness to make a quit attempt over time (B = −19.86, *p* = 0.02) and assisting them to quit by providing a treatment or making a referral for a treatment (B = −19.93, *p* = 0.04). (Table 2).

## 4. Discussion

Previous research has demonstrated the success of Taking Texas Tobacco Free (TTTF), a comprehensive tobacco-free workplace intervention aiming to increase the use of the five A’s related to smoking cessation within substance use treatment centers in Texas, U.S. [45,52]. The results from the current study expand on these findings by highlighting how provider factors aggregated to the organization level were associated with changes in the use of the five A’s in the context of the program implementation within substance use treatment centers in Texas, U.S. Specifically, centers that started off with lower average levels of (1) providers’ beliefs they should address patients’ tobacco use or (2) providers’ self-efficacy for delivering tobacco use assessments experienced greater changes in (1) asking patients about their smoking and assisting them with a smoking quit attempt through intervention or referral and (2) assessing their interest in quitting, respectively.

In essence, centers with providers that had the most to learn and master regarding addressing patients’ tobacco use seemingly embraced the new ideas and skills to a greater extent than centers with providers who were already better informed and trained. Although suppositional, it may be that less equipped substance use treatment centers were more ‘primed’ to gain new information and training relative to centers that had previously been exposed to this information. Likewise, organizations that started with high levels of these factors may have ceiling or threshold effects. If so, the results highlight a need to potentially tailor trainings for individual substance use treatment centers based on the pre-implementation data to minimize the redundancy with prior exposures, increase the diversity of the training experiences provided, and ultimately facilitate greater intervention uptake and more positive outcomes. However, it is important to note that novelty effects may wane over time. Consequently, longer-term follow-up may be needed within substance use treatment centers implementing a tobacco-free workplace program to ensure that changes in addressing patients’ tobacco use are sustained over time. This is especially relevant given the high rates of provider turnover in these settings [63,64,65,66]. The current study also did not have any statistically significant findings related to advising individuals to quit smoking or arranging follow-up with patients to see how the quit attempt progressed (or to encourage another quit attempt). Although both of these evidence-based intervention steps increased in occurrence by post-implementation (~14% for advise and ~27% for arrange), more work is needed to understand the center-level mechanisms that drove these changes.

It is important to highlight that it was not the changes in provider beliefs regarding the importance of addressing tobacco use in non-nicotine substance use disorder treatment and self-efficacy that predicted changes in their use of three of the five A’s; rather, it was their pre-implementation beliefs and self-efficacy. Importantly, prior research supports that many providers have misconceptions that addressing conventional cigarette smoking during non-nicotine substance use treatment is harmful to patients and can impede provider intervention [21]. Moreover, behavior change theory supports that building self-efficacy for a behavior increases its likelihood of occurrence [62]. The failure for gains over time in the accuracy of provider beliefs about cessation intervention and in building self-efficacy for tobacco use assessment conduct may suggest that other organizational factors were more influential to providers’ smoking intervention behavior change. For example, the support of center leadership for the program may be more important for behavioral change than individual-level changes in provider education and skill-building (aggregated to the center level).

Providers in substance use treatment centers commonly report several barriers to screening and intervention in patients’ smoking, including, but not limited to, a lack of time and training [22,23,24,25,26,27,28,29,30]. The current study found that substance use treatment centers with more provider-reported barriers to routine smoking cessation intervention delivery at pre-implementation experienced greater gains in assessing patients’ interest in quitting smoking. Likewise, centers that reported greater reductions in barriers by post-implementation had statistically significant (greater) gains in assessing patients’ interest in quitting smoking and assisting patients with a quit attempt through intervention or referral. This latter finding is notable, as the reduction in reported barriers over time across all centers was less than one (SD < 1). Thus, interventions that aim to reduce barriers to the provision of smoking cessation care in substance use treatment settings may be essential to encourage organizational behavior changes. This may be particularly important within organizations that report many barriers at pre-implementation. Furthermore, the findings support that substance use treatment centers with providers reporting many barriers to providing smoking cessation care should not be deterred from participating in workplace interventions to build a tobacco intervention capacity, as they may gain the most from their participation.

### 4.1. Limitations and Strengths

There are a few limitations and strengths that should be mentioned. First, the intervention was implemented in a limited number of substance use treatment centers in Texas. The results may differ based on the number of centers enrolled and the geographic locations of these centers. However, conducting analyses at the organizational level provides aggregate data, reducing the possibility that high turnover rates (a known problem within substance use treatment centers [63,64,65,66]) may introduce bias. Second, changes over time in the independent variables were generally small (~4%), which may affect the statistical power necessary to detect associations. However, power analyses indicated that an effect size of 0.60 or greater (i.e., a large effect size) would be sufficient power for the study’s sample size, given α = 0.05 and a power of 0.80. Additionally, center-level covariates were not controlled for in the analytic models. Moreover, our procedures did not include the collection of the number of providers employed at the centers when the pre- and post-implementation surveys were distributed by the program champions, precluding an accurate response rate calculation. Likewise, although we encouraged the centers to have all providers attend the tobacco trainings we offered and were as accommodating as possible in our scheduling, we are unable to accurately estimate the proportion of providers (versus employees overall) who attended these trainings. Lastly, the outcome was change in the use of the five A’s; however, because the data collection was anonymous, we could not calculate the individual provider-level changes. In this work, we elected to use cluster-level averages, which has a precedent in the literature for similarly structured datasets [68,69] and which recognizes the potential for influence at the center level (e.g., organization-level factors such as normative beliefs, values, culture, and policies and/or communities of practice among providers [57]) in shaping the overall patient care provision. Future works would benefit from the explicit assessment of multilevel factors during program implementation, use of the growing arsenal of implementation science measures available in the literature, and the application of multilevel modeling techniques to the resulting data with attention to appropriate covariate inclusion to further advance the field.

Despite the limitations, there are a number of strengths to be mentioned. First, the study examined factors that are associated with the use of the five A’s, which are an efficacious intervention for tobacco use. Moreover, the organizational-level factors were examined to inform future iterations of the intervention as a way to increase the intervention efficacy and bridge the research-to-translation gap. Furthermore, the study focused on an outcome and population that is often overlooked: tobacco use in individuals with a non-nicotine substance use disorder.

### 4.2. Future Directions

Given that the Socioecological Model posits many levels of influence on behavior [56], future research may focus on further disentangling other organization-level influences (e.g., organizational readiness for change [52]) on the provision of smoking cessation care within substance use treatment centers. Moreover, as there was a lack of statistically significant findings relating to the intervention components of advising individuals to quit smoking and arranging a follow-up, despite the demonstrated changes in them over time, future research may want to focus on these intervention components to better understand the possible influences. Additionally, there may be other organizational-level interventions not part of this tobacco-free workplace intervention that might be used to complement educational and skill-building efforts to build a capacity for tobacco interventions. For example, programming a ‘hard stop’ in the electronic health record system that requires addressing the five A’s may increases providers’ provision of brief screening and intervention for smoking with their patients [70,71,72]. This factor was not included or evaluated in the present study; the authors’ anecdotal experience was that many participating substance use treatment centers still used paper records and/or did not have the capability, funding, or interest to change their electronic health records in this regard.

## 5. Conclusions

This study found that organization-level beliefs in addressing tobacco use, self-efficacy for conducting a tobacco use assessment, and barriers to providing cessation care to patients in substance use treatment centers in Texas, U.S. were statistically associated with the use of three of the five A’s (ask, assess, and assist) for smoking cessation after the implementation of a comprehensive tobacco-free workplace program. These results suggest that centers that had providers with less desirable pre-implementation beliefs, lower levels of self-efficacy in conducting tobacco use assessments, and more barriers to the provision of routine smoking cessation care experienced greater gains in the tobacco intervention capacity through the tobacco-free workplace program implementation. Furthermore, centers with greater reductions in barriers over time gained a larger tobacco intervention capacity from the tobacco-free workplace program implementation. The findings from this study contribute to advancing implementation science, which has increasingly been focused on understanding the factors that underlie organizational change. Ultimately, the goal of implementation science is to reduce the research-to-practice translational gap in the most efficient manner [73,74]. The findings from this study also provide future avenues to explore, such as organizational factors that may influence an increase in advising patients to quit smoking and arranging a follow-up within substance use treatment programs participating in a comprehensive tobacco-free workplace program.

## Figures and Tables

**Table 1:** Center-level average percentages/means of pre-implementation variables and the changes by post-implementation.

	Pre-Implementation	Changes (Post–Pre)
	Mean or Percent (SD)	
Independent Variables		
Belief in Addressing Tobacco Use	86.52% (11.70%)	3.90% (10.37%)
Self-Efficacy *	67.98% (21.44%)	4.14% (21.16%)
Barriers	1.60 (0.67)	−0.73 (0.76)
Dependent Variables		
Ask	76.25% (20.29%)	13.99% (19.05%)
Advise	72.02% (21.25%)	14.16% (18.54%)
Assess	79.80% (23.08%)	8.78% (24.85%)
Assist	54.28% (28.62%)	26.24% (28.18%)
Arrange	46.23% (29.70%)	26.84% (24.89%)

Notes. SD = standard deviation. *N* = 15 substance use treatment centers (* = analyses for self-efficacy were assessed for the 11 centers that had data on this variable). The number of providers completing pre-implementation surveys within the centers ranged from 3 to 65 (total pre-implementation *N* = 259 for all centers combined). The number of providers completing post-implementation surveys within the centers ranged from 1 to 50 (total post-implementation *N* = 194 for all centers combined). More information on center-specific characteristics and survey completion can be found in our prior work [52]. Self-efficacy relates to conducting tobacco use assessments, and barriers relate to those for the routine provision of tobacco use disorder care.

**Table 2:** Associations between organizational-level factors and changes in the use of the five A’s for smoking cessation from before to after a comprehensive tobacco-free workplace implementation.

Provider Behaviors	Independent Variable	β	SE	*p*-Value
**Pre-Implementation**				
Change in Ask	Belief in Addressing Tobacco Use	**−0.91**	**0.37**	**0.03**
	Self-Efficacy *	−0.45	0.22	0.08
	Barriers	11.02	7.30	0.15
Change in Advise	Belief in Addressing Tobacco Use	−0.77	0.38	0.07
	Self-Efficacy *	−0.10	0.23	0.68
	Barriers	0.71	7.70	0.93
Change in Assess	Belief in Addressing Tobacco Use	−0.14	0.59	0.82
	Self-Efficacy *	**−0.93**	**0.27**	**0.01**
	Barriers	**23.40**	**8.02**	**0.01**
Change in Assist	Belief in Addressing Tobacco Use	**−1.52**	**0.52**	**0.01**
	Self-Efficacy *	−0.40	0.44	0.38
	Barriers	14.58	10.98	0.21
Change in Arrange	Belief in Addressing Tobacco Use	−0.30	0.58	0.61
	Self-Efficacy *	−0.51	0.38	0.20
	Barriers	17.34	9.15	0.08
**Changes (post–pre)**				
Change in Ask	Belief in Addressing Tobacco use	0.38	0.50	0.46
	Self-Efficacy *	0.09	0.27	0.76
	Barriers	−10.56	6.26	0.12
Change in Advise	Belief in Addressing Tobacco use	0.53	0.47	0.28
	Self-Efficacy *	0.19	0.23	0.44
	Barriers	−5.07	6.58	0.46
Change in Assess	Belief in Addressing Tobacco use	0.42	0.65	0.53
	Self-Efficacy *	−0.29	0.41	0.50
	Barriers	**−19.86**	**7.15**	**0.02**
Change in Assist	Belief in Addressing Tobacco use	1.15	0.68	0.12
	Self-Efficacy *	0.37	0.45	0.44
	Barriers	**−19.93**	**8.61**	**0.04**
Change in Arrange	Belief in Addressing Tobacco use	0.87	0.62	0.18
	Self-Efficacy *	−0.35	0.40	0.41
	Barriers	−16.22	7.84	0.06

Notes. SE = standard error. *N* = 15 substance use treatment centers. * *n* = 11 centers. Bolded text indicates statistically significant results at *p* < 0.05. Self-efficacy relates to conducting tobacco use assessments, and barriers relate to those for the routine provision of tobacco use disorder care.

## Data Availability

The data presented in this study are available on request from the corresponding author. The data are not publicly available due to confidentiality/privacy agreements with the funders.
